# Diagnostic Value of Serum Apolipoprotein B100 Combined With Hippocampal Volume in Alzheimer's Disease

**DOI:** 10.1002/brb3.70066

**Published:** 2024-09-30

**Authors:** Dandan Zhang, Jing Wu, Guoqiang Ren, Yi Wang, Hang Xu, Siyuan Chen, Xuezhong Li, Xiaopeng Chen

**Affiliations:** ^1^ Department of Neurology The Affiliated People's Hospital of Jiangsu University Zhenjiang Jiangsu People's Republic of China; ^2^ Department of Radiology The Affiliated People's Hospital of Jiangsu University Zhenjiang Jiangsu People's Republic of China; ^3^ Department of Neurology The People's Hospital of Jurong City Jurong Jiangsu People's Republic of China

**Keywords:** Alzheimer′s disease, apolipoprotein B100, diagnostic value, hippocampal volume

## Abstract

**Purpose:**

To explore the diagnostic value of serum apolipoprotein B100 (Apo B100) combined with hippocampal volume in Alzheimer's disease (AD).

**Methods:**

A total of 59 AD patients and 59 healthy subjects were selected. The Mini‐Mental State Examination (MMSE) was used for neuropsychological assessment. Blood glucose and serum lipid levels were detected by biochemical analyzer. Polymerase chain reaction (PCR) was used to detect apolipoprotein E (Apo E) ε3/ε4 genotypes in the plasma. Hippocampal volume was calculated using Slicer software. Independent‐sample *t* test or Mann–Whitney *U* test were used to compare the levels of various indicators between the two groups. Spearman's correlation analysis was used to analyze the correlation between each level. The receiver operating characteristic curve (ROC) was plotted, and the area under the curve (AUC) was calculated to compare the diagnostic efficacy of individual and combined detection of serum Apo B100 levels and hippocampal volume in AD.

**Results:**

Compared with the healthy control group, the levels of serum total cholesterol (TC), low‐density lipoprotein (LDL), Apo B100, and plasma Apo E ε3/ε4 were higher in the AD group, and serum high‐density lipoprotein (HDL) level was lower in the AD group (both *p *< 0.05). The hippocampal volume in the AD group was lower than in the control group (*p *< 0.01). The serum Apo B100 level was negatively correlated with MMSE score (*r = *−0.646), whereas hippocampal volume was positively correlated with MMSE score (*r *= 0.630). ROC curve analysis showed that the AUC of the combined serum Apo B100 level and hippocampal volume for AD was higher than that of either alone (AUC = 0.821, *p *< 0.01).

**Conclusion:**

Serum Apo B100 level is elevated, and the hippocampal volume is reduced in AD patients. The combined detection of the two has a higher diagnostic efficiency for AD than other alone and has the potential to become an important indicator for the diagnosis of AD in the future.

## Graphical abstract

To explore the diagnostic value of serum apolipoprotein B100 (Apo B100) combined with hippocampal volume in Alzheimer's disease (AD). The receiver operating characteristic curve (ROC) was plotted, and the area under the curve (AUC) was calculated to compare the diagnostic efficacy of individual and combined detection of serum Apo B100 levels and hippocampal volume in AD. ROC curve analysis showed that the AUC of the combined serum Apo B100 level and hippocampal volume for AD was higher than that of either alone (AUC = 0.821, *p* < 0.01).

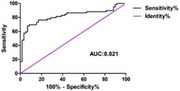

## 1 Introduction

Alzheimer's disease (AD) is a common cause of dementia, and its main clinical manifestation is a progressive decline in cognitive and behavioral functions. Its neuropathological features include abnormal deposition of amyloid β (Aβ) and intracellular hyperphosphorylation of tau protein in the neurofibrillary tangles (Scheltens et al. 2021). Studies have shown that polymorphisms in the apolipoprotein E (Apo E) gene are a major risk determinant of late‐onset AD (Yamazaki et al. 2019). Apo E has also been shown to cause susceptibility to AD by disrupting serum lipid levels (Tang et al. 2019). Numerous clinical and epidemiological studies have shown that lipid metabolism disorders are closely related to the risk, pathogenesis, and progression of AD (Tang et al. 2019). A previous study showed that apolipoprotein B (Apo B) is not associated with AD (Houlden et al. 1995). However, recent studies have shown that the elevated level of Apo B in the cerebrospinal fluid (CSF) of AD patients is closely associated with pathological changes in tau in the early stage of the disease and is highly expressed in the entorhinal cortex and hippocampal gyrus (Picard et al. 2022). Hu et al. (2020) found that Apo B may be a potential biomarker for preclinical AD, which attracted our attention. This study mainly explored the changes of serum apolipoprotein B100 (Apo B100) in AD patients and its relationship with hippocampal volume, as well as the diagnostic value of serum Apo B100 combined with hippocampal volume in AD to discover biomarkers that can be used for the diagnosis of AD and provide a basis for the diagnosis of AD.

## 2 Methods

### 2.1 Patients

A total of 59 (27 male and 32 female, mean age: 71.22 ± 6.89 years) AD patients were selected from Affiliated People's Hospital of Jiangsu University between September 2021 and September 2023 as the research target. Furthermore, 59 older individuals (30 male and 29 female, mean age: 71.34 ± 6.02 years) who underwent physical examinations during the same period were selected as the control group. All subjects met the diagnostic criteria for the “AD clinical syndrome” established by the American Institute of Aging and the AD Association in 2018 (Jack et al. 2018). Inclusion criteria for the control group were as follows: (1) The clear absence of cognitive impairment and other severe physical diseases; (2) Mini‐Mental State Examination (MMSE) score 27–30 points. This study was approved by the Ethical Committee of the Affiliated People's Hospital of Jiangsu University (ethics number: SH2022073) and conducted in accordance with the Declaration of Helsinki (as revised in 2013). All participants in the present study completed and submitted informed consent.

### 2.2 Data Collection

#### 2.2.1 General Clinical Data Collection

The general clinical data of subjects, including age, sex, smoking, drinking, hypertension, diabetes, coronary heart disease, and education, were collected.

#### 2.2.2 Blood Glucose, Serum Lipid Levels, and Plasma Apo E ε3/ε4

Briefly, 5 mL of anticoagulant and nonanticoagulant fasting venous blood was collected from all subjects in the morning. The biochemical analyzer was used to detect serum levels of fasting blood glucose (FBG), total cholesterol (TC), triglycerides (TG), high‐density lipoprotein (HDL), low‐density lipoprotein (LDL), apolipoprotein A1 (Apo A1), and Apo B100. Polymerase chain reaction (PCR) was used to detect Apo E ε3/ε4 genotypes in plasma. The above indicators were tested by laboratory professionals.

#### 2.2.3 Cognitive Assessment

All patients were evaluated using a neuropsychological scale by a neurologist in a quiet state. Cognitive function was evaluated using the MMSE scale, with a total score of 30 points. Illiteracy ≤17 points, primary school education ≤20 points, and high school and above education ≤26 points were considered to denote cognitive impairment, whereas MMSE > 26 points was considered to denote normal cognition.

#### 2.2.4 Hippocampal MRI and Hippocampal Volume

A Siemens Skyra 3.0T MRI scanner, equipped with a 20‐channel coil, was used to perform brain and hippocampus MRI examination. The selected patients were placed in a supine position and asked to remain calm during testing. Coronal T2‐weighted (T2WI) structural images were obtained with the following parameters: a voxel size, 0.4 × 0.4 × 0.2 mm^3^; slice thickness, 2 mm; matrix, 256 × 256; time of repetition (TR), 4000 ms; and time of echo (TE), 84 ms. A specialist neurologist measured the patient's hippocampal volume in a blinded manner. The regions of interest (ROI) of the bilateral hippocampus were manually delineated layer by layer in T2WI by 3D‐slicer software. The software calculated the left and right hippocampal volumes, respectively, and the total hippocampal volume was the sum of the left and right hippocampal volumes.

### 2.3 Statistical Analysis

All statistical analysis and plotting were performed in SPSS 25.0 (IBM Corporation, Armonk, NY, USA) and GraphPad Prism 9.0 software. The measurement data were first tested for normal distribution. Normal distributions were expressed as ($\bar{x}$ ± *s*), and the comparison between the two groups was performed by *t* test. Nonnormally distributed data were expressed as the median and inter quartile range [*M* (*P*25, *P*75)], and the Mann–Whitney *U* test was used to compare the two groups. Countable data were expressed as percentages (%), and the chi‐squared test was used for between group comparisons. Spearman's correlation coefficient was used for correlation analysis. The diagnostic efficacy of serum Apo B100 levels and hippocampal volume alone and in combination was assessed by plotting the receiver operating characteristic (ROC) curve and calculating the area under the curve (AUC) and calculating the sensitivity and specificity. A two‐sided *p* value of <0.05 was considered to indicate statistically significant differences.

## 3 Results

### 3.1 Comparison of General Clinical Data Between the Two Groups

Comparison between the two groups in terms of age, sex, smoking, drinking, hypertension, diabetes, coronary heart disease, and education showed no statistically significant differences (*p *> 0.05). The MMSE score of the AD group was lower than that of the control group, and the difference was statistically significant (*p *< 0.01) (Table 1).

**TABLE 1:** Comparison of general clinical data between the two groups.

	AD group (*N* = 59)	Control group (*N* = 59)	*p*
Age (years)	71.22 ± 6.89	71.34 ± 6.02	0.921
Male (%)	27 (45.76%)	30 (50.84%)	0.580
Education (years)	8 (4,9)	7 (5,10)	0.778
Smoking (%)	12 (20.34%)	14 (23.73%)	0.657
Drinking (%)	17 (28.81%)	19 (32.20%)	0.689
Hypertension (%)	26 (44.07%)	24 (40.68%)	0.709
Coronary heart disease (%)	7 (11.86%)	4 (6.78%)	0.342
Diabetes (%)	12 (20.33%)	10 (16.95%)	0.636
MMSE score	18.00 (15.00,22.00)	29.00 (28.00,29.00)	0.000

*Notes*: Values are the mean ± SD or number of cases.

Abbreviations: AD, Alzheimer's disease; MMSE, Mini‐Mental State Examination.

### 3.2 Comparison of Blood Glucose and Serum Lipid Levels Between the Two Groups

The serum levels of serum FBG, TG, and Apo A1 did not show statistically significant differences between the two groups (*p *> 0.05). The serum TC, LDL, and Apo B100 levels were higher in the AD group than the healthy control group, and the differences were statistically significant (*p *< 0.05, *p *< 0.01, and *p < *0.01, respectively). The serum HDL level was lower in the AD group (*p *< 0.05), and the plasma Apo E ε3/ε4 was higher in the AD group (*p *< 0.05) than the control group (Table 2).

**TABLE 2:** Comparison of blood glucose and serum lipid levels between the two groups.

	AD group (*N* = 59)	Control group (*N* = 59)	*p*
FBG (mmol/L)	5.10 (4.50,5.70)	4.90 (4.30,5.70)	0.561
TG (mmol/L)	1.37 (0.78,1.99)	1.28 (1.03,2.01)	0.534
TC (mmol/L)	4.15 (3.74,4.92)	3.83 (3.46,4.43)	0.011
HDL (mmol/L)	1.21 ± 0.35	1.38 ± 0.43	0.023
LDL (mmol/L)	2.34 (2.05,3.01)	2.22 (1.81,2.45)	0.006
Apo A1 (g/L)	1.38 (1.18,1.61)	1.35 (1.27,1.53)	0.741
Apo B100 (g/L)	1.32 (1.13,1.41)	1.01 (0.83,1.23)	0.000
Apo E ε3/ε4 (%)	15 (25.42%)	6 (10.17%)	0.030

*Notes*: Values are the mean ± SD or number of cases.

Abbreviations: AD, Alzheimer's disease; Apo A1, apolipoprotein; Apo B100, apolipoprotein B100; Apo E ε3/ε4, apolipoprotein E ε3/ε4; FBG, fasting blood glucose; HDL, high density lipoprotein; LDL, low density lipoprotein; TC, total cholesterol; TG, triglycerides.

### 3.3 Comparison of Hippocampal Volumes Between the Two Groups

The left, right, and total hippocampal volume of patients in the AD group were smaller than those in the control group, with statistically significant differences (*p *< 0.01) (Table 3).

**TABLE 3:** Comparison of hippocampal volumes between two groups.

	AD group (*n* = 59)	Control group (*n* = 59)	*p*
Left hippocampal volume (cm^3^)	2.24 ± 0.40	2.62 ± 0.37	0.000
Right hippocampal volume (cm^3^)	2.38 ± 0.43	2.76 ± 0.39	0.000
Total hippocampal volume (cm^3^)	4.62 ± 0.82	5.39 ± 0.74	0.000

*Notes*: Values are the mean ± SD or number of cases.

Abbreviation: AD, Alzheimer's disease.

### 3.4 Correlation Analysis of Serum Apo B100 Level, Total Hippocampal Volume, and MMSE Score

**FIGURE 1:**
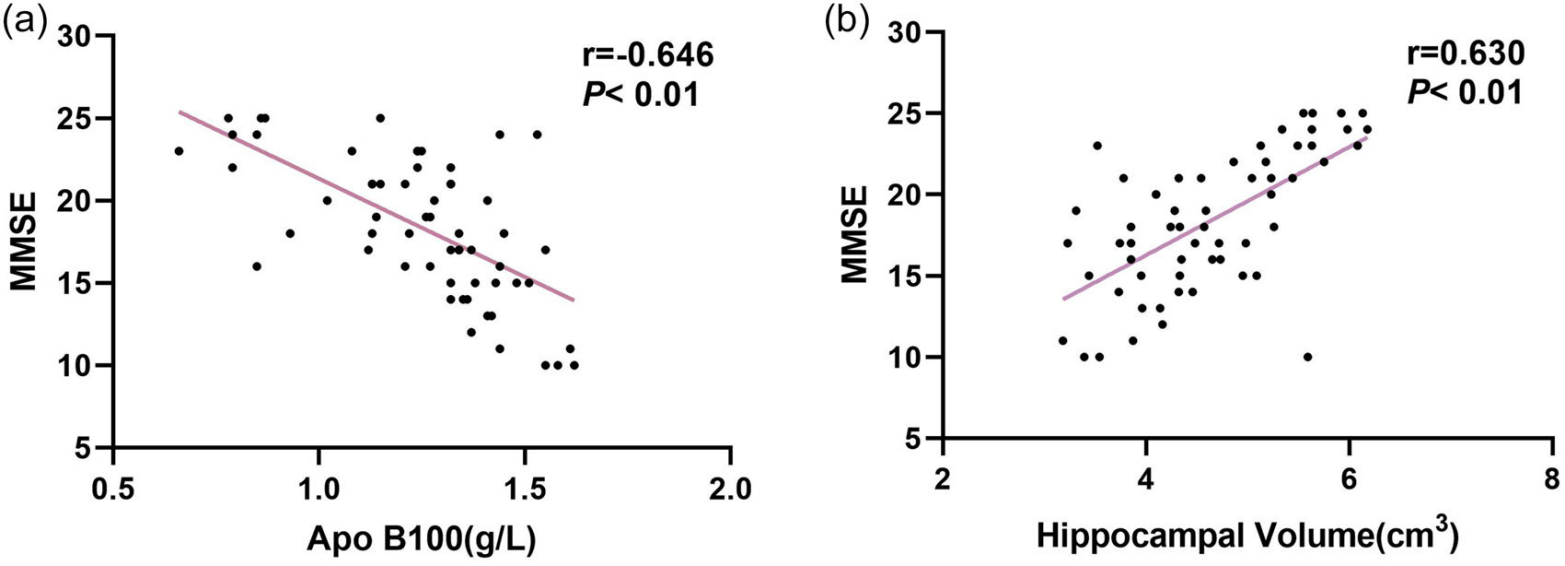
Correlation analysis of serum Apo B100 level, total hippocampal volume, and MMSE score in patients with AD. (a) Negative correlation between MMSE score and Apo B100 (*r* = −0.646, *p* < 0.01). (b) Positive correlation between MMSE score and hippocampal volume (*r* = 0.630, *p* < 0.01). Apo B100, apolipoprotein B100; AD, Alzheimer's disease; MMSE, Mini‐Mental State Examination.

The serum Apo B100 level was negatively correlated with MMSE score (*r *= −0.646, *p *< 0.01), whereas hippocampal volume was positively correlated with MMSE score (*r *= 0.630, *p *< 0.01) (Figure 1).

### 3.5 Diagnostic Efficacy of Serum Apo B100 Level and Total Hippocampal Volume Alone and in Combination for AD

**FIGURE 2:**
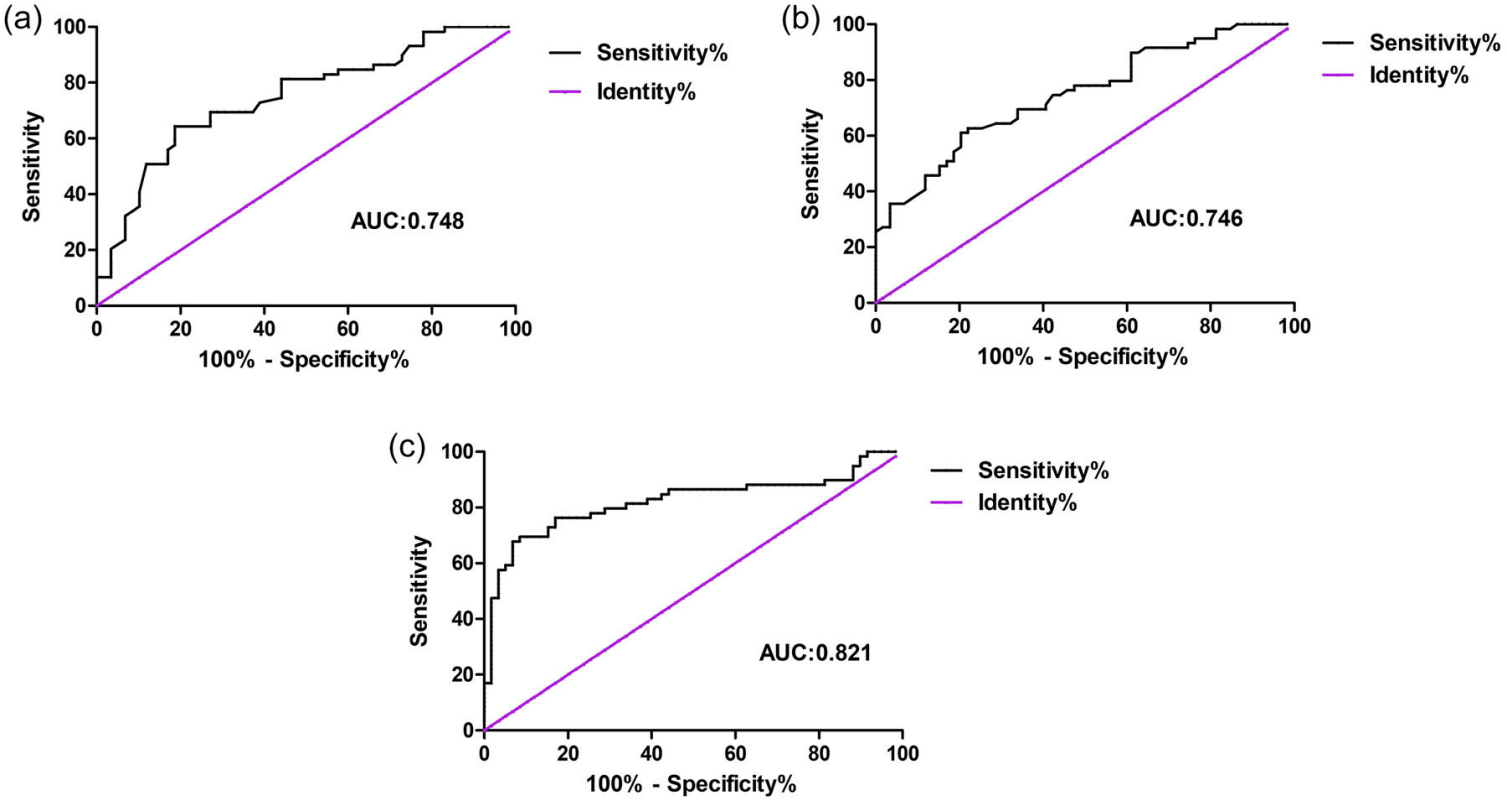
Receiver operating characteristic (ROC) curves for the diagnosis of AD. (a) ROC curve of serum Apo B100, (AUC = 0.748, sensitivity = 64.4%, specificity = 81.4%). (b) ROC curve of hippocampal volume (AUC = 0.746, sensitivity = 62.7%, specificity = 78.0%). (c) ROC curve of combined serum Apo B100 and hippocampal volume (AUC = 0.821, sensitivity = 69.5%, specificity = 91.5%). AD, Alzheimer's disease; Apo B100, apolipoprotein B100; AUC, area under the curve.

The ROC curve analysis showed that the AUC of serum Apo B100 level in diagnosing AD was 0.748, with a sensitivity of 64.4% and specificity of 81.4%. The AUC of the total hippocampal volume in diagnosing AD was 0.746, with a sensitivity of 62.7% and specificity of 78.0%. The AUC of the combined diagnosis of serum Apo B100 and hippocampal volume for AD was 0.821, with a sensitivity of 69.5% and a specificity of 91.5% (Figure 2).

## 4 Discussion

Clinical studies on lipidomics and metabolomics have found that AD patients can experience changes in various lipid class levels (Tang et al. 2019). Our study showed that the serum TC, LDL, and Apo B100 levels in the AD group were higher than those in the control group, whereas the serum HDL level in the AD group was lower (*p *< 0.05). This may be because of serum lipid levels affecting amyloid production, oxidative stress, neuroinflammation, and/or other reasons in AD patients (Yin 2023). Our study showed that the plasma Apo E ε3/ε4 in the AD group was higher than that in the control group (*p *< 0.05). At present, research on Apo E in AD is maturing, and a large number of previous studies have shown that Apo E gene polymorphisms are the major genetic risk factors for the onset of AD (Asante, Louie, and Yassine 2022; Xu et al. 2020; Zverova et al. 2018). The results of this study once again validate this conclusion.

Accumulating evidence suggests that there is a strong relationship between changes in the central nervous system (CNS) lipid homeostasis and degenerative diseases of the nervous system, especially AD, which may be related to the formation of Aβ amyloid plaques. Apo B is produced in the liver and intestine to assist LDL in transporting cholesterol throughout the body. Apo B can be detected in the CSF, which may play a role in maintaining CNS lipid homeostasis (Picard et al. 2022). Although Apo B is detected in CSF, it is not produced at this site. A possible explanation is a partial leakage of the blood‐brain barrier (BBB) for unknown reasons, allowing plasma lipoproteins and other macromolecular components to enter more easily into the interior of the brain (Chang et al. 2017). Takechi et al. (2010) found that a diet high in fatty acids exhibited the accumulation of Apo B, and that could damage the integrity of the BBB, which leads to the blood‐brain transmission of Apo B. In the late 1900s, it was shown that the Apo B immunoreactivity was associated with brain amyloid and neurofibrillary tangles in AD patients (Namba, Tsuchiya, and Ikeda 1992). Moreover, the serum Apo B level was significantly higher in AD patients than normal controls (Caramelli et al. 1999). However, more recent research tended to focus on Apo E rather than Apo B being associated with AD (Houlden et al. 1995). Furthermore, in recent years, studies on the basis of gene sequencing, human brain, and animal studies had revealed that Apo B may be involved in the pathogenesis of AD (Hoyk et al. 2018; Wingo et al. 2019). A study on genetic sequencing of AD patients showed a strong correlation between rare genetic coding variants of Apo B and AD (Wingo et al. 2019), which could promote the formation of amyloid plaques and have a direct correlation with AD risk (Agarwal and Khan 2020). A recent study by Picard et al. (2022) on human CSF Apo B levels and AD CSF biomarkers (Aβ amyloid and tau protein levels) showed that CSF Apo B levels were increased in patients with AD and significantly correlated with tau protein levels in the entorhinal cortex area, fusiform gyrus, and para‐hippocampal gyrus, which were associated with decreased longitudinal visuospatial cognition. Meanwhile, some studies also showed elevated serum Apo B levels in AD patients (Agarwal and Khan 2020), which others suggested that Apo B played different roles in different stages of AD. In the preclinical stage of AD, Apo B has a protective relationship with CSF biomarkers, and the serum Apo B level was reduced. However, in the clinical stage of AD, the serum Apo B level is elevated, which is likely related to factors such as disease stage, lipid metabolism status, and genetics. Currently, there is no consistent conclusion (Hu et al. 2020). Some animal experiments also verified the correlation between Apo B and AD, considering its involvement in the processes of tau protein hyperphosphorylation, synaptic dysfunction, and cognitive impairment in AD (Hoyk et al. 2018; Lénárt et al. 2012). A study found that elevated levels of Apo B could be detected in the cortex and hippocampus of the AD mouse model, and amyloid plaque formation could be seen in the cerebral cortex, hypothalamus, and hippocampus of brain tissue sections, which is a typical characteristic of AD (Lénárt et al. 2012). Hoyk et al. (2018) detected extensive apoptosis of cortical and hippocampal neurons in a transgenic mouse model overexpressing human Apo B100, with a significant reduction in the number of dendritic spines in hippocampal neurons and increased permeability in hippocampal BBB, accompanied by structural changes, considered related to cognitive impairment in AD. This study showed that the serum Apo B100 level in the AD group was significantly higher than that in the control group. Moreover, compared with other serum lipid indices, the serum Apo B100 level in the two groups was the most statistically significant, which was significantly negatively correlated with the MMSE score, indicating that Apo B100 was involved in AD progression. The reason may be that Apo B100 was involved in amyloid deposition and neurofibrillary tangles in the brain areas (especially the hippocampus) of AD patients, leading to impaired synaptic function, thereby affecting cognitive function (Hoyk et al. 2018; Namba, Tsuchiya, and Ikeda 1992).

The hippocampus is an important component of the limbic lobe and a key region for learning and memory. In the early stages of AD, the hippocampus can exhibit extremely rapid tissue loss, which was reportedly associated with significant short‐term memory decline in AD patients (Rao et al. 2022). Neuroimaging studies in AD (Hari et al. 2022) have shown that patients diagnosed with dementia have significant structural changes in their brains, mainly manifested as a reduction in medial temporal lobe volume, particularly in the hippocampus volume, which was the most significant structural imaging change correlated with memory scores. A study by Chong et al. (2021) showed that hippocampal atrophy was associated with CSF phosphorylated tau protein in AD patients. Another study by Rao et al. (2023) showed that the smaller the hippocampal volume, the more severe the cognitive impairment in AD patients. In our study, hippocampal volume reduction in the AD group was significant and positively correlated with MMSE score, which was consistent with previous studies. This suggested that the reduction in hippocampal volume may be used as an imaging marker for the diagnosis of AD.

We further analyzed the diagnostic efficacy of serum Apo B100 and hippocampal volume alone and in combination for the diagnosis of AD. Using binary logistic regression method combined with serum ApoB level and hippocampal volume to construct a regression model, our results showed that the AUC for diagnosing AD was 0.821 and had the highest specificity (95% confidence interval: 0.740–0.902), which was greater than the AUC for diagnosing AD with a single indicator. Hence, the combination of the two had more important value as a biomarker for the diagnosis of AD.

This study has some limitations. First, the sample size is small, and future studies with larger sample sizes are needed to confirm our conclusions. Second, serum ApoB level can dynamically change over time and are affected by diet, medication, and daily lifestyle. We did not evaluate the effects of diet, medication, and other factors on serum ApoB level. Third, we only discussed the diagnostic efficacy of Apo B100 combined with hippocampal volume in AD, which is still unclear how Apo B100 affects the reduction of hippocampal volume. This is worthy of further investigation to improve the understanding of AD pathogenesis.

## 5 Conclusion

In summary, serum Apo B100 level is elevated, and the hippocampal volume is reduced in AD patients. The combined detection of the two has a higher diagnostic efficiency for AD and has the potential to become an important indicator for the diagnosis of AD.

## Author Contributions

**Dandan Zhang**: investigation, writing—original draft, data curation, and writing—review. **Jing Wu**: visualization. **Guoqiang Ren**: project administration. **Yi Wang**: investigation. **Hang Xu**: data curation. **Siyuan Chen**: methodology. **Xuezhong Li**: project administration and validation. **Xiaopeng Chen**: Revise manuscript, writing—review and editing, and supervision.

## Conflicts of Interest

The authors declare no conflicts of interest.

### Peer Review

The peer review history for this article is available at https://publons.com/publon/10.1002/brb3.70066.

## Data Availability

The data that support the findings of this study are openly available in piaoyicxp at https://doi.org/10.1002/brb3.70066, reference 23.
